# Facet-Destructive Cervical Tumoral Calcinosis Causing Myelopathy in Systemic Sclerosis: A Case Report and Literature Review

**DOI:** 10.7759/cureus.115202

**Published:** 2026-08-26

**Authors:** Kishor Kumar, Ira Sun, Joseph Feng, Shiong Wen Low, Chun Peng Goh

**Affiliations:** 1 Neurosurgery, Medicover Hospitals, Hyderabad, IND; 2 Neurosurgery, Ng Teng Fong General Hospital, Singapore, SGP

**Keywords:** cervical myelopathy, scleroderma, spinal stenosis, systemic sclerosis, tumoral calcinosis

## Abstract

Tumoral calcinosis (TC) is a rare disorder characterized by periarticular ectopic calcium deposition, with spinal involvement being exceptionally uncommon. We report a rare case of facet-destructive cervical TC causing progressive cervical myelopathy in a patient with diffuse systemic sclerosis and review the current literature on its pathogenesis, diagnosis, and management.

A 51-year-old woman with diffuse systemic sclerosis presented with progressive neck pain, worsening cervical kyphosis, and cervical myelopathy. Computed tomography and magnetic resonance imaging demonstrated multilobulated calcific masses involving the posterior elements and facet joints from C3 to C6, resulting in severe cervical canal stenosis. She underwent C3-6 decompressive laminectomy with posterior instrumented fusion. Intraoperatively, the lesions had extensively replaced the bilateral lateral masses and posterior elements from C3 to C5 and were densely adherent to the dura, precluding complete excision. Adequate decompression was achieved through careful internal debulking and lateral release of the calcified masses, with intraoperative ultrasonography confirming satisfactory decompression. Postoperatively, her neck pain resolved and neurological function improved progressively; independent ambulation was regained by six months.

Although rare, spinal TC should be considered in the differential diagnosis of calcified spinal lesions, particularly in patients with connective tissue disease. Surgical decompression with stabilization remains the treatment of choice for patients presenting with neurological compromise.

## Introduction

Tumoral calcinosis (TC) is an uncommon disorder characterized by ectopic deposition of calcium phosphate crystals within periarticular soft tissues [1]. First described by Inclán in 1943, TC is broadly classified into primary normophosphatemic, primary hyperphosphatemic, and secondary forms according to its underlying pathophysiology [2]. Secondary TC is most commonly associated with chronic kidney disease, trauma, and connective tissue disorders such as systemic sclerosis. Although periarticular involvement is typical, spinal disease is exceedingly rare, with only isolated cases reported in the literature [3-11]. Here, we report a rare case of facet-destructive cervical TC causing progressive cervical myelopathy in a patient with diffuse systemic sclerosis. We also discuss the diagnostic challenges, operative considerations, and current evidence regarding the management of spinal TC.

## Case presentation

A 51-year-old woman with diffuse systemic sclerosis complicated by interstitial lung disease, pericardial effusion, dysphagia, and osteoporosis presented with a one-year history of progressive neck pain, worsening cervical kyphosis, and gait instability, with acute deterioration in left lower limb weakness. Serological investigations demonstrated a high antinuclear antibody (ANA) titre of 1:640 (negative <1:80), strongly positive anti-Scl-70 antibodies at 81 U (>50 U considered strongly positive), and positive anti-Ro-52 and anti-RNP/Sm antibodies.

Neurological examination revealed a spastic gait with asymmetric lower-limb weakness, most pronounced on the left, with Medical Research Council grade 2/5 hip flexion. Sensory examination demonstrated reduced sensation over the left medial and lateral calf, first dorsal web space, and heel. Her modified Japanese Orthopaedic Association (mJOA) score was 12/18 (3/5 upper extremity motor, 3/7 lower extremity motor, 3/3 sensory, and 3/3 bladder function). Serum investigations showed normal corrected calcium (2.32 mmol/L; reference range 2.10-2.55 mmol/L) and phosphate (1.28 mmol/L; 0.75-1.50 mmol/L) levels, with a low serum creatinine level of 29 µmol/L (45-90 µmol/L) and elevated urea at 31 mmol/L (3.5-7.2 mmol/L).

**Figure 1:**
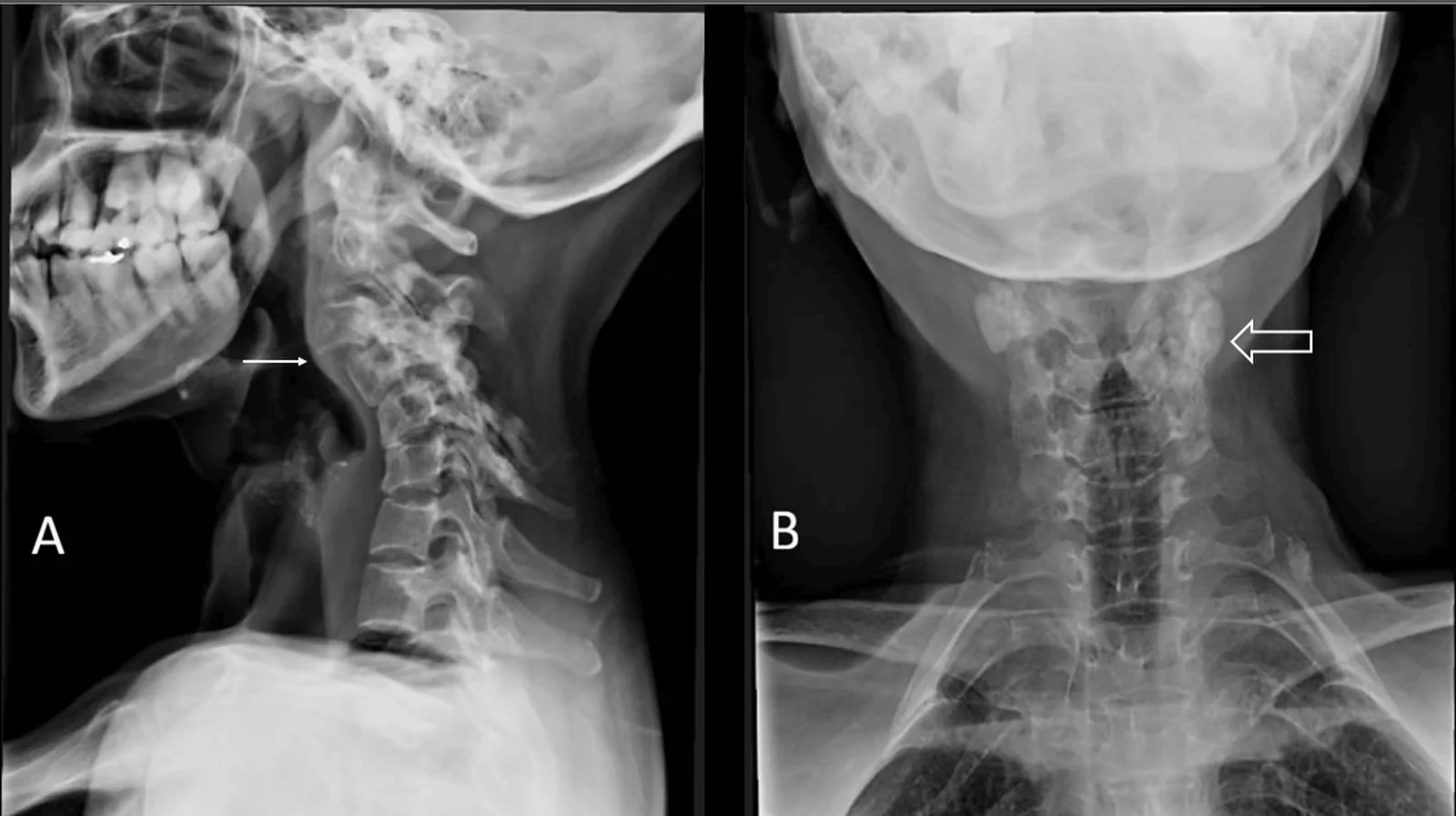
Cervical radiographs: (A) lateral and (B) anteroposterior views demonstrating Grade II anterolisthesis of C3 on C4 and C4 on C5, with an associated S-shaped cervical curvature (solid arrow). Extensive soft tissue calcifications were identified involving the posterior elements from C3 to C6 (hollow arrow).

**Figure 2:**
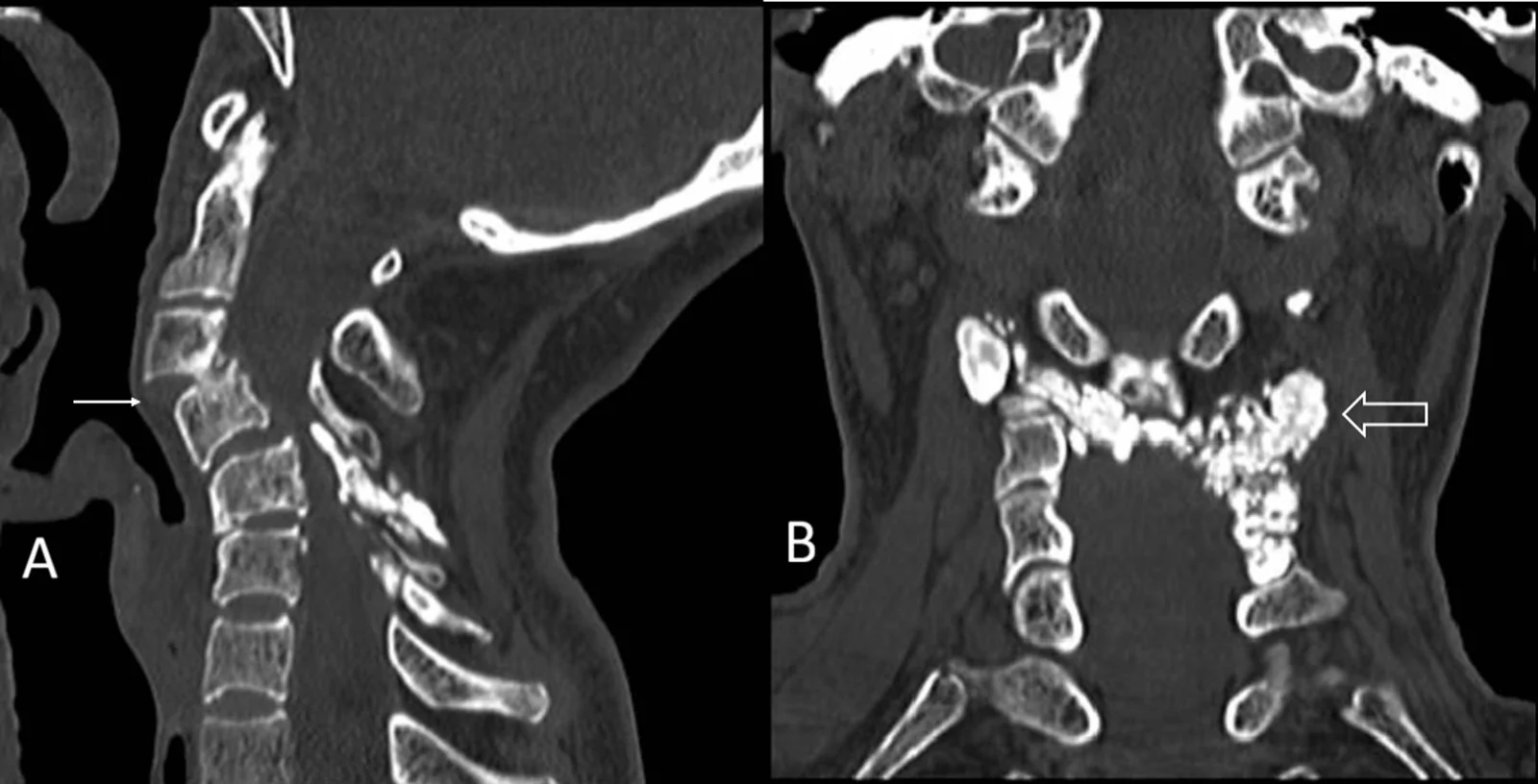
Computed tomography scan: (A) mid-sagittal and (B) coronal views demonstrating multilobulated calcified masses centered on the C3-C6 facet joints and posterior elements (hollow arrow), with associated facet destruction and anterolisthesis at C3-4 and C4-5 (solid arrow).

**Figure 3:**
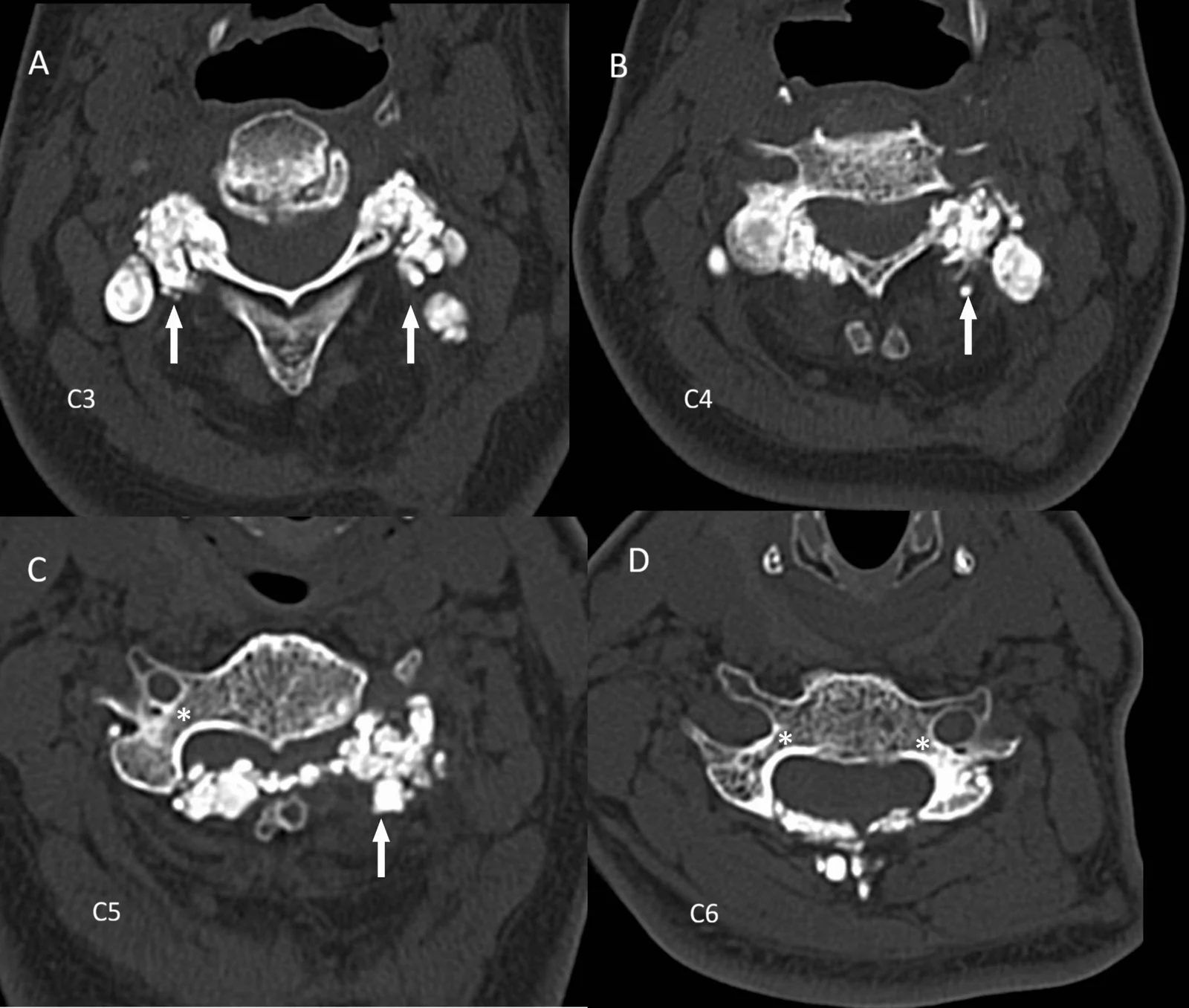
Representative axial cuts of cervical spine CT: (A and B) extensive destruction of both C3 and C4 lateral masses (arrows). (C) Destruction of the left C5 lateral mass (arrow) with an intact right pedicle (asterisk). (D) Replacement of C6 lamina with calcinosis with intact pedicles on both sides (asterisks).

**Figure 4:**
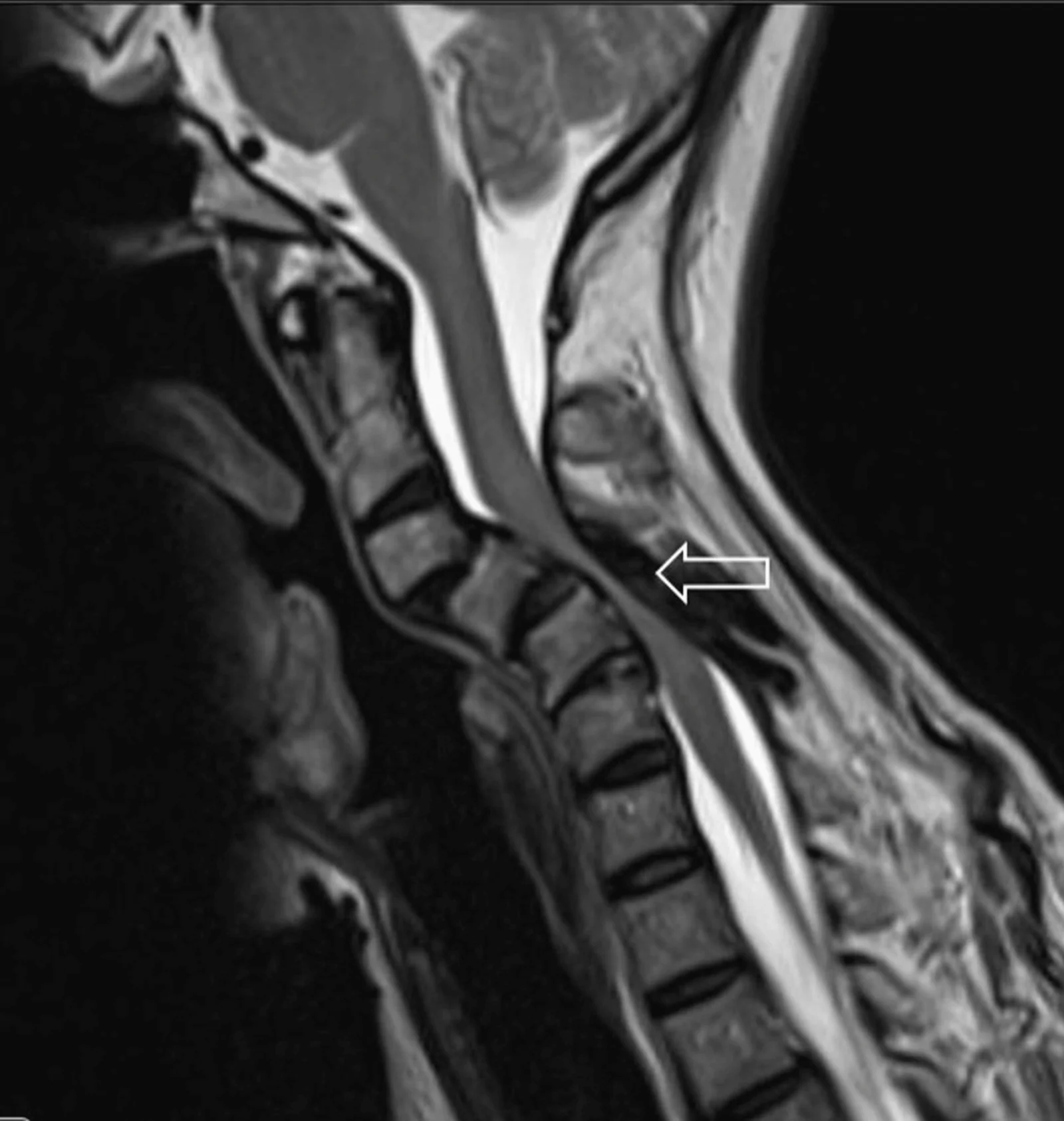
Cervical spine MRI demonstrating severe cord compression with myelomalacia at the C3-4 to C5-6 levels (arrow).

Cervical radiographs demonstrated Grade II anterolisthesis of C3 on C4 and C4 on C5, with an associated S-shaped cervical curvature. Extensive soft tissue calcifications were identified involving the posterior elements from C4 to C6 (Figure 1). Computed tomography demonstrated multilobulated calcified masses centered on the C3-C6 facet joints and posterior elements, with associated facet destruction and anterolisthesis at C3-4 and C4-5 (Figures 2, 3). MRI demonstrated severe cervical canal stenosis with marked cord compression at C3-4 to C5-6 levels (Figure 4). The radiological differential diagnosis included brown tumour secondary to hyperparathyroidism, calcified meningioma, epidural lipomatosis with calcification, and dystrophic calcification associated with autoimmune disease. Given her progressive neurological deficits, she underwent C3-6 laminectomy with C2 undercutting and posterior instrumented fusion.

**Figure 5:**
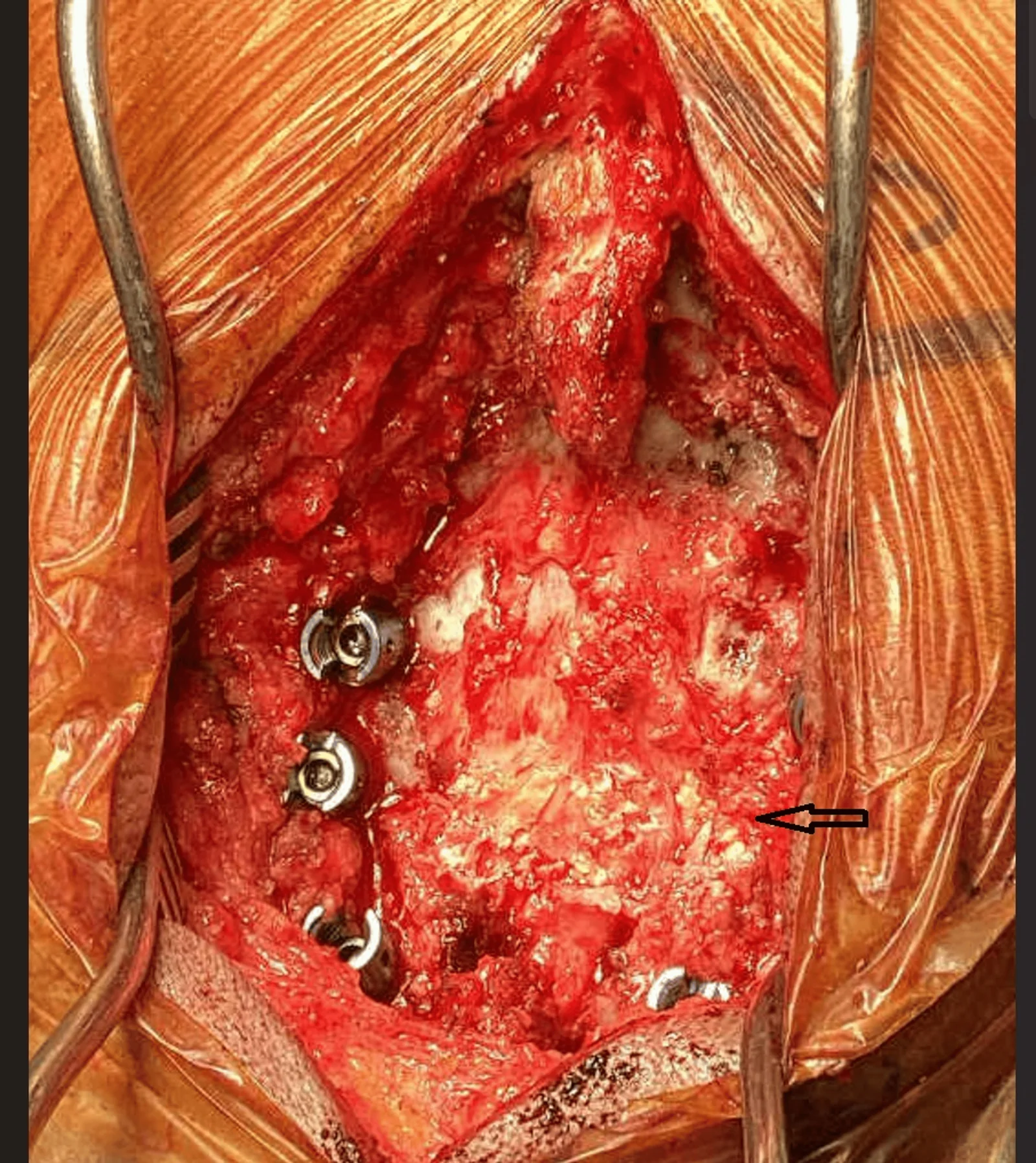
Intraoperative image showing friable, chalk-like calcified material (arrow) replacing much of the posterior elements and lateral masses.

**Figure 6:**
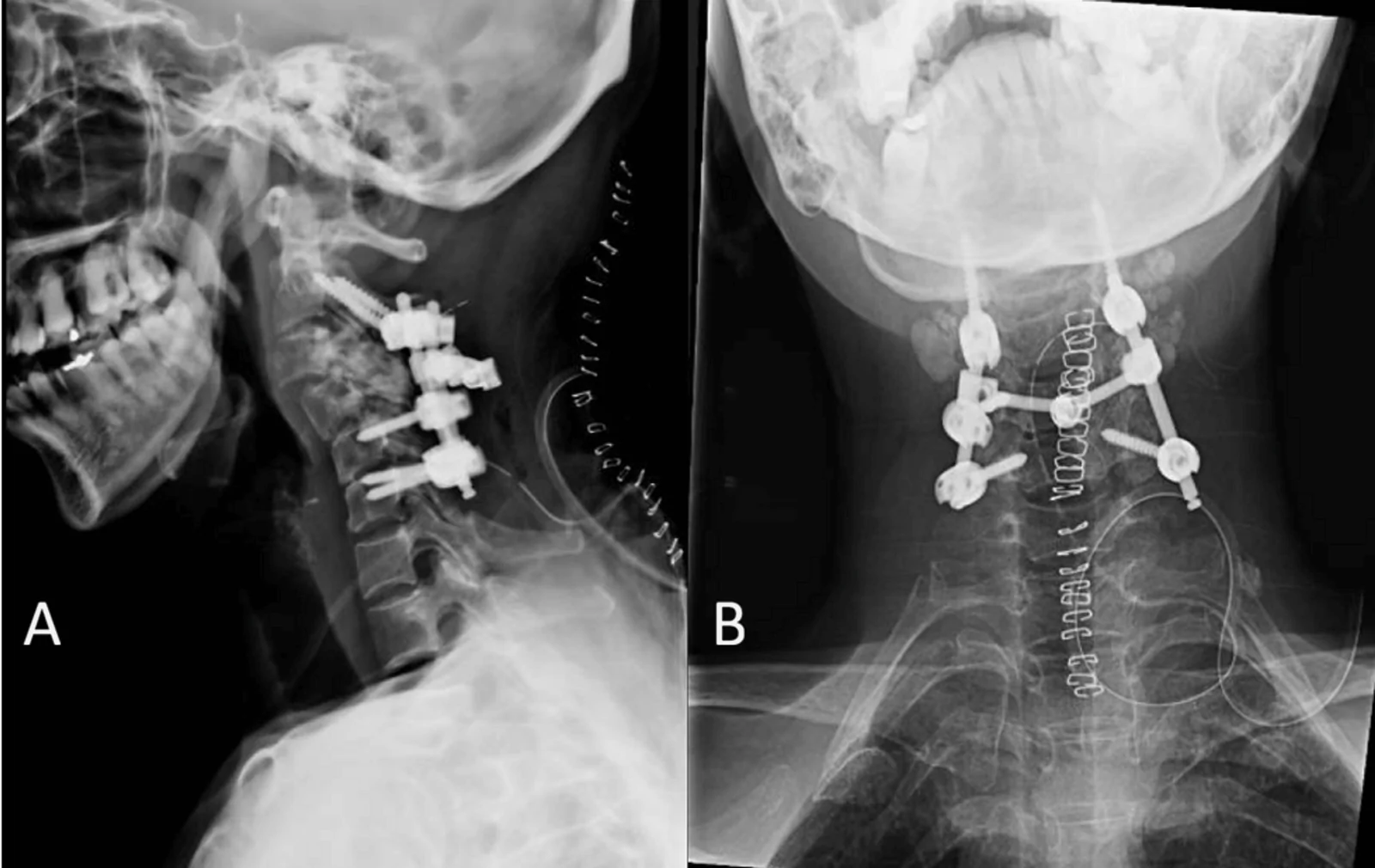
Cervical radiographs: (A) lateral and (B) anteroposterior views. Posterior cervical instrumentation consisted of bilateral C2 pars screws, a single right C5 screw, and bilateral C6 pedicle screws.

Intraoperatively, the lesions consisted of friable, chalk-like calcified material replacing much of the posterior elements and lateral masses, resulting in a marked loss of osseous fixation points (Figure 5). Instrumentation therefore consisted of bilateral C2 pars screws, a right C5 screw, and bilateral C6 pedicle screws (Figure 6).

Dense adherence of the calcified masses to the dorsal dura precluded complete excision, necessitating subtotal resection followed by careful spinal cord decompression without breaching the dura. Intraoperative ultrasonography confirmed restoration of cerebrospinal fluid flow dorsal to the spinal cord. Histopathological examination demonstrated extensive dystrophic calcification within fibrous tissue, consistent with secondary TC, without evidence of malignancy or granulomatous inflammation.

Postoperatively, her neck pain resolved and lower limb strength improved progressively to Medical Research Council grade 4/5. Following inpatient rehabilitation, she regained independent ambulation. Her mJOA score had improved to 14/18 (4/5 upper extremity motor, 4/7 lower extremity motor, 3/3 sensory, and 3/3 bladder function). At the one-year follow-up, she remained neurologically stable without new deficits.

## Discussion

TC most commonly affects the shoulder, hip, and elbow regions, whereas spinal involvement is exceptionally uncommon [1,2]. When the spine is affected, patients typically present with axial pain, progressive myelopathy, radiculopathy, or mechanical instability secondary to progressive facet and posterior element destruction [4,6]. In patients with systemic sclerosis who develop progressive cervical myelopathy, spinal TC should therefore be included in the differential diagnosis despite its rarity.

CT remains the imaging modality of choice for characterizing the extent of calcification, osseous destruction, and facet joint involvement [6], whereas MRI is invaluable for assessing spinal cord compression and neural element compromise [6,7]. CT and MRI provide complementary information for diagnosis, operative planning, and differentiation from other calcified spinal lesions [7].

Histopathological examination is critical for confirming the diagnosis and excluding other mimics, such as neoplasms or infectious causes [7]. In the present case, crystal composition analysis was not performed, representing a diagnostic limitation. Although the histopathological findings were consistent with tumoral calcinosis, the absence of crystal analysis precluded further characterization of the calcific deposits and definitive exclusion of other crystal deposition disorders, such as calcium pyrophosphate dihydrate (CPPD) deposition disease.

Surgical decompression with stabilization remains the treatment of choice for symptomatic spinal TC associated with neurological compromise [6,8,10]. Extensive destruction of the posterior elements may substantially reduce available fixation points, necessitating modification of standard instrumentation strategies [2]. In our patient, dense dural adherence precluded complete excision. Rather than pursuing aggressive dissection with its attendant risk of dural injury, decompression was achieved through internal debulking and lateral release of the calcified masses, allowing re-expansion of the thecal sac without excessive manipulation of the adherent dura. Intraoperative ultrasonography provided a valuable adjunct for confirming adequate decompression despite residual adherent calcified tissue.

Published reports have consistently demonstrated favorable neurological outcomes following surgical decompression in appropriately selected patients [5,6,8,10,11]. A review of the literature suggests that cervical involvement is the most common site of spinal TC, with progressive myelopathy and radiculopathy being the predominant presenting symptoms [11]. Surgical decompression, with or without instrumented fusion, generally results in good neurological recovery when significant neural compression is present [5]. However, extensive posterior element destruction and dense dural adherence, as encountered in our patient, have been only infrequently described, highlighting the technical challenges associated with operative management of advanced disease.

Medical therapy plays an adjunctive role in secondary or uremic TC by correcting the underlying metabolic derangement [12-15]. Treatment strategies include phosphate binders, calcimimetics, low-calcium dialysate, and intensified hemodialysis. In selected patients, parathyroidectomy [12] or renal transplantation [13] may result in regression or complete resolution of the lesions.

Our case highlights several important features. First, spinal TC associated with systemic sclerosis remains exceptionally rare. Second, extensive destruction of the cervical facet joints and lateral masses created significant challenges for spinal instrumentation. Finally, dense dural adherence necessitated a modified decompression strategy, with intraoperative ultrasonography serving as an adjunct to confirm adequate decompression. These operative findings may provide useful technical insights for surgeons managing similarly complex cases.

## Conclusions

Although spinal involvement is rare, tumoral calcinosis should be considered in the differential diagnosis of calcified spinal lesions, particularly in patients with systemic sclerosis or other connective tissue disorders. Early recognition, accurate radiological diagnosis, and timely surgical decompression with stabilization are essential for maximizing neurological recovery and preventing irreversible spinal cord injury.
